# Intrinsic Capacity in Older Adults: Recent Advances

**DOI:** 10.14336/AD.2021.0818

**Published:** 2022-04-01

**Authors:** Yaru Zhou, Lina Ma

**Affiliations:** Department of Geriatrics, Xuanwu Hospital, Capital Medical University, National Clinical Research Center for Geriatric Disorders, Beijing, China.

**Keywords:** intrinsic capacity, healthy aging, integrated care, frailty, resilience, older adults

## Abstract

The global increase in the aging population is expected to result in a shift from disease-centered to function-centered approaches in response to intensive aging. Thus, the World Health Organization (WHO) has proposed a novel concept, intrinsic capacity (IC), which refers to the combination of one’s physical and mental abilities. The IC framework comprises cognition, mobility, psychological, vitality, and sensory functions. WHO also issued the *Guidelines on Integrated Care for Older People (ICOPE)* in 2017 and the *Handbook: Guidance on person-centred assessment and pathways in primary care*in 2019 to provide recommendations for community-level interventions and clinical practice, respectively. Recently, studies on the assessment of IC and verification of IC measurement have been proliferating. In this study, we reviewed the recent advances in IC research with older adults.

The global population is rapidly aging [1]. According to the World Health Organization (WHO), older adults aged ≥ 60 years will account for 12% in 2015 to 22% in 2050 (2 billion) of the world’s population (www.who.int/news-room/fact-sheets/detail/ageing-and-health). To formulate public health strategies in response to the aging population, WHO has proposed the concept of healthy aging as the process of developing and maintaining the functional ability required for the healthy life of older adults (apps.who.int/iris/handle/10665/186463). Functional ability depends on intrinsic capacity (IC) and the environment, as well as the interaction between them. IC refers to the sum of an individual's physical and mental abilities. Evidence shows that focusing on the IC of older adults is more effective than focusing on specific chronic diseases [2-4]. Therefore, WHO proposed *Guidelines on Integrated Care for Older People (ICOPE)* for the maintenance of IC (apps.who.int/iris/handle/10665/258981). However, research on IC assessment and on the development of interventions to improve IC is still in its infancy. This article reviews the progress of research on IC as well as its implications for clinical work.

## Healthy Aging and Intrinsic Capacity

### Intrinsic capacity

The *World Report on Ageing and Health* by WHO proposed the novel concept of healthy aging, as the process of developing and maintaining the functional ability that enables well-being in old age (apps.who.int/iris/handle/10665/186463). Accordingly, functional ability is described as “the health-related attributes that enable people to be and to do what they have reason to value” [5]. Furthermore, IC refers to the sum of the physical and mental capacities of an individual, determining the functional ability combined with environmental factors and their interaction (apps.who.int/iris/handle/10665/186463). Older adults can achieve higher quality of life in their later years, when they are within a suitable environment and have reached the peak of each health phase, thus reducing the burden of the society. The existing care model for older adults is to predict and respond to diseases by targeting specific disease markers. Wang et al [6] reviewed that the shift from disease-centered care to IC has major implications for nursing practice in older hospitalized adults. However, the new WHO care model of healthy aging involves a longitudinal observation of the individual's trajectory, with the goal of implementing active and personalized interventions that improve older adults’ IC and functional ability (apps.who.int/iris/handle/10665/186463).

### Research on the evaluation of intrinsic capacity

IC is a strong predictor for health outcomes from the perspective of function. There are several complex IC indicators; therefore, which of these can be used specifically to evaluate the overall physical and mental state of an individual? Cesari [7] used the International Classification of Functioning, Disability and Health framework as a base, combined it with available evidence, and identified the five IC domains (locomotion, vitality, cognition, psychological, and sensory) as the key to controlling and maintaining the IC of older adults, thereby permitting subsequent evaluation of IC. Similarly, Beard [8] assessed walking speed, chair-stand test, balance, grip strength, forced expiratory volume, blood assay, sensory, cognitive function, and sleep in the English Longitudinal Study on Ageing (ELSA) and found that five subfactors (psychological, sensory, cognitive, vitality, and locomotor) formed a structure to better predict future functioning, which is consistent with the findings of Cesari. A recent Chinese study also validated this structure using the same method as in ELSA [9]. Based on these studies, researchers from different countries have evaluated and verified the predictive value of the decline of IC and its domains in older adults.

**Table 1:** Longitudinal studies on intrinsic capacity in older adults

Author/year	Country/ region	Study	Follow-up time	Research setting	Participants and sample size	IC assessment	Main outcome measure	Results
Beard et al. 2019	United Kingdom	ELSA	2 years	Community	≥ 60 years N = 2560	Walking speed, chair-stand test, balance, grip strength, FEV, blood assay, sensory, cognitive, affect, sleep	ADL, IADL	IC had a direct relationship with the outcome. Multimorbidity had an independent direct relationship with incidence loss of ADLs but not IADLs, and it also operated through IC. IC mediated more of the indirect effect of personal characteristics on incidence loss of ADLs and IADLs than multimorbidity.
Liu et al. 2021	Beijing, China	-	2 years	Community	> 75 years N = 212	MMSE, SPPB, MNA-SF, GDS-15, vision and hearing evaluation	Katz ADL index Falls	Orientation and memory impairment were associated with a higher probability of functional decline. The impaired chair stand test, weight loss, and little interest in doing things were significantly associated with falls.
Yu et al. 2021	Hong Kong, China	The MrOS and MsOS (Hong Kong) study	7 years	Community	≥ 65 years N = 3736	Cognition: MMSE Locomotor: walking speed, chair stands, dynamic balance Vitality: grip strength, adiposity to muscle ratio, body fat/ASM Sensory: binocular visual acuity, stereopsis Psychological: GDS-15	IADL	IC predicted incident IADL limitations directly. The direct effect of IC on IADL was larger than the direct effect of the number of chronic diseases.
Beard et al. 2021	China	CHARLS	2 years	Community	≥ 60 years N = 7643	Walking speed, the chair-stand test, balance, grip strength, FEV, hemoglobin, hearing and vision impairments, episodic memory, intact mental status, affect and sleep quantity/quality.	ADL, IADL	IC predicted the declining performance in ADL and IADL both directly and indirectly. The direct effect was much larger than the indirect effect of IC through multimorbidity. Both multimorbidity and IC independently predicted the declining ADLs and IADLs. Personal characteristics predicted declining ADLs and IADLs both directly and indirectly through IC and multimorbidity.
Charles et al. 2020	Belgian	SENIOR	3 years	Nursing home	N = 604	Cognition: MMSE Locomotion: SPPB Sensory: the self-report Strawbridge questionnaire Vitality: abdominal circumference, handgrip strength, MNA Psychosocial: depression (3-point Likert scale) Fatigue (two questions from the C ES-D)	Death, falls, autonomy decline	A one-unit increase in the balance performance and nutrition score decreased the probability of death (by 12%) and the risk of fall (by 4%). No association was found between IC and repeated falls. Low scores in nutrition were associated with a higher probability of autonomy decline.
Zeng et al. 2021	Zhejiang, China	-	1 year	Hospital	≥ 60 years N = 329	Cognition: MMSE Locomotion: B-POMA, 4-m gait speed test Sensory: self-reported hearing and vision status Vitality: handgrip strength, MNA-SF Psychological: GDS-15	ADL, IADL, mortality	Low MMSE scores at admission predicted 1-year new ADL and IADL dependency. No significance was observed between IC domains and mortality. Higher IC score at admission was associated with decreased risks of 1-year new ADL and IADL dependency, and mortality.

Abbreviations: IC, Intrinsic Capacity; ELSA, the English Longitudinal Study on Ageing; FEV, Forced expiratory volume; ADL, activities of daily living; IADL, instrumental activities of daily living; MMSE, the Mini-Mental-State-Examination; SPPB, Short Physical Performance Battery Test; MNA-SF, Mini-Nutritional Assessment Short Form; GDS-15, Geriatric Depression Scale-15; CES-D, Epidemiologic Studies Depression scale; MrOS and MsOS (Hong Kong), the Mr. Osteoporosis and Ms Osteoporosis (Hong Kong) study; ASM, appendicular skeletal muscle mass; CHARLS, the China Health and Retirement Longitudinal Study; SENIOR, Sample of Elderly Nursing home Individuals: an Observational Research; MNA, Mini Nutritional Assessment; B-POMA, balance subscale of Tinetti Performance-Oriented Mobility Assessment.

A general IC index, rather than simple effects of different subdomains added together, may provide better predicted value. Thus far, no global IC index has been validated for clinical or research purposes [14]. Therefore, more research on the validation of IC concepts and constructs is required, along with further quantitative evaluation of IC and its domains in different settings.

## Intrinsic capacity, frailty, and physical resilience

### IC and frailty

IC, represents the amount of resources available to a person over a lifetime, is a dynamic concept, and its trajectory can provide information about the entire lifespan to clinical and public health activities, at an individual or a national level [15]. Clinicians can detect deviations from the norm before clinical manifestations, evaluate the effectiveness of interventions, and take preventive measures to achieve healthy aging. As a novel concept, IC has plenty of distinctions as well as commonalities and connections with frailty.

Frailty is a geriatric syndrome in which the gradual decline of an individual's physiological system makes the individual more susceptible to stressors and increases the risk of adverse health outcomes [15]. Furthermore, frailty is a condition that appears before the onset of disability, most often associated with the latter phase of life [16].

IC and frailty can be viewed as two sides of the same coin [10], where the former is an individual’s reserve of ability, whereas the latter a hindrance that grows with aging. However, the two concepts are not mutually exclusive. Belloni [15] argued that IC, to some extent, can be considered as an evolution of the concept of frailty. The two concepts are complementary; monitoring IC can support the concurrent evaluation of individual frailty. Assessing the IC of a frail individual can also prove valuable, such as by resulting in the development of an individual, personalized care plan based on an individual's thoughts and priorities. Robledo [17] argued that the IC score acts as a determinant of frailty, pre-frailty, or robustness among adults, suggesting that the IC indices were significantly associated with frailty. Thus, further studies are needed to untangle the intricate relationship between IC and frailty.

## IC and physical resilience

Physical resilience is a new concept in the field of geriatrics introduced by the National Institute on Aging (NIA)[18]; it is defined as the ability to recover from physically or psychologically traumatic events [19]. Consistent with IC, physical resilience also focuses on positive health attributes, and it targets function instead of diseases [20]. Chhetri [20] demonstrated that IC is a major determinant of physical resilience via physiological reserve. Therefore, we can assume that appropriate actions that improve IC can also improve physical resilience. However, more evidence and verification are required on this topic. Table 2 presents the comparisons among IC, frailty, and physical resilience.

**Table 2:** Comparisons of intrinsic capacity, frailty, and physical resilience.

	Intrinsic capacity	Frailty	Physical resilience
Concept	A composite of all mental and physical capacities.	A clinical syndrome that reflects a state of increased vulnerability to multiple adverse outcomes.	An ability to recover from physically or psychologically traumatic events.
Characteristic	Positive attributes	Negative effects	Positive attributes
Context	Healthy aging	Opposite of successful aging	Successful aging
Trajectory	Throughout the lifespan	Later phase of life during the downhill trajectory before disability occurs	Throughout the lifespan but a response after external stressors
Indicators/ Measurement Approaches	Mobility: balance, chair stand, gait speed Cognition: time orientation, three-word recall Vitality: grip strength, BMI Psychological: low energy/fatigue, depression Sensory: vision, hearing	Biological factors: individual factors, nutrition, medical conditions, physical abilities Psychological factors: cognition, depression, emotional regulation, motivation, stress appraisal Social factors: community, social status, social connections, family/friend support	Phenotypes: frailty, robustness, fatigability Age discrepancy: biological vs. chronological age Trajectory: after prior or experimental stressors

Abbreviations: BMI, body mass index.

## Intrinsic capacity screening tools

The 2019 *Integrated Care for the Elderly (ICOPE)-Guidance on person-centred assessment and pathways in primary care* introduced by WHO prepared a quick and easy screening tool for IC. The WHO ICOPE screening tool for IC was validated among Chinese adults [21], which indicated that the proportion of decline in mobility, cognition, vitality, hearing, vision, and psychology was 25.3%, 46.8%, 16.2%, 15.4%, 11.7%, and 12.0%, respectively; furthermore, lower IC scores were associated with increasing age, slow walking speed, poor grip strength, and frailty [21]. This was the first study to validate the ICOPE screening tool, suggesting that the tool proposed by WHO can be effective in identifying people exhibiting decline in IC. A 2-year study with 212 adults aged ≥ 75 years validated this prediction for functional decline and fall [10]. Similarly, the Multidomain Alzheimer Preventive Trial, for older 759 adults (70-89 years old) with memory issues, reported that the frequencies of cognitive decline, limited mobility, malnutrition, visual impairment, hearing loss and depressive symptoms were 52.2%, 20.2%, 6.6%, 18.1%, 56.2%, and 39%, respectively, using the ICOPE screening tool [22]. Thus, we require additional validation studies with larger sample sizes that explore pragmatic ways to implement the ICOPE screening tool within various contexts.

## Intrinsic capacity biomarkers

IC reflects an individual's biological aging process, and its evaluation may provide an innovative mechanism for encouraging adults to adopt healthy lifestyles. Identification of potential biomarkers of IC decline may provide simple and objective measurements of an individual's biological age and health status and may guide the development of strategies to minimize IC decline, even delay progress toward disability [23]. However, research on the identification of IC biomarkers is rare.

In a 5-year follow-up study, participants with elevated homocysteine or C-reactive protein (CRP) levels demonstrated a decrease in IC, and this decrease was more significant among those exhibiting higher CRP levels combined with hyperhomocysteinemia [24]. Moreover, participants with deteriorating IC reported significantly higher levels of plasma N-terminal pro-B-type Natriuretic Peptide (NT-proBNP) than those with normal IC, and the former were associated with abnormal mobility, hearing, vision, and psychological function [25]. In addition, increased serum tumor necrosis factor receptor 1 (TNFR1) levels were independently associated with reduced IC, suggesting that chronic inflammation may be the basis for decline in IC [26]. Based on this evidence, some studies have proposed lifestyle interventions [27-29] or supplements of omega-3 (ω-3) polyunsaturated fatty acid (PUFA)— known for its anti-inflammatory effects [30-34]—to mitigate the effects on cognitive impairment [27-30,34] and other domains [31-33]. However, a 3-year study with French community-dwelling participants, free of major neurocognitive disorders, reported no improvement in IC after undergoing ω-3 supplementation and multidomain lifestyle intervention [31-33]. Furthermore, no existing study has examined the effect on the decline in overall IC [35]. Therefore, further investigation is required on the biomarkers for IC decline and the development of interventions to delay overall IC decline.

## Integrated care for older people (ICOPE)

### ICOPE Handbook-Guidance on person-centred assessment and pathways in primary care

To address the adverse events due to the decline of IC, WHO issued the *ICOPE* guidelines to manage IC decline in 2017 (apps.who.int/iris/handle/10665/258981). Thirteen recommendations were provided, including reducing mobility loss, reducing malnutrition, maintaining visual and hearing ability, preventing cognitive decline and depression, management of age-related conditions and falls, and provision of support to caregivers. WHO also published the *Handbook: Guidance on person-centred assessment and pathways in primary care* in 2019 to help community-health and care workers implement the ICOPE recommendations (apps.who.int/iris/handle/10665/326843). It outlines a healthcare pathway for managing critical health conditions associated with IC decline. Personalized care plans integrate strategies to reverse, delay, or prevent further decline.

### The worldwide implementation of the ICOPE guidelines

Researchers from various countries have made innovative efforts to implement the ICOPE guidelines. The INStitute for Prevention healthy agIng and medicine REjuvenative (INSPIRE) initiative in France aimed to build a bio-resource research platform for healthy aging by gathering biological, clinical, and digital resources to identify markers of aging, age-related diseases, and IC evolution [36,37]. Scientists plan to conduct a large-scale clinical survey of ICOPE using the ICOPE screening tool with older adults every 4 months for 10 years. INSPIRE will demonstrate the feasibility of screening and assessing IC level in primary care services, as well as conduct remote and long-term monitoring of this population’s IC [38]. In collaboration with the INSPIRE program, Tavassoli [37] examined IC in 962 older adults from Occitania, in accordance with the 5-step ICOPE guidelines, and found that most older adults demonstrated a decline in at least one IC subdomain, with decline in vision, hearing, and cognitive function being most commonly reported. The ICOPE Monitor, a new technology derived from the INSPIRE program, has been developed to assess individuals’ IC [36]. The ICOPE application is another tool developed by WHO [39]. These two applications are readily available free of charge in the Apple or Android Store [40]. China has proposed the “Medical and old-aged care integration model” as a strategy and implemented it as a policy in the recent years. China has incorporated the novel concept of healthy aging, put forth by WHO, into its existing care model and modified it to be people-centered so that older adults can both achieve healthy aging and enjoy their old life better [41]. Further research on the implementation of abovementioned new platform and technology within clinical settings and households is required.

## Conclusion

Active management of the aging population has become a challenge. IC, the sum of all physical and mental functions, is of great value in predicting subsequent care dependence rather than the single or multiple disease investigation approach. To this end, WHO issued the ICOPE guidelines and handbook in 2017 and 2019, respectively, to aid primary healthcare workers, who provide care for older adults, using recommendations for the management and care of decline in different IC subdomains, as well as a care pathway for identifying and screening IC in order to assess, manage, and provide appropriate support to caregivers. Future research should focus on the development of interventions and integration of novel concepts in clinical and routine care provision for older adults.

## References

[1] Beard JR, Officer A, de Carvalho IA, Sadana R, Pot AM, Michel J, et al. (2016). The World report on ageing and health: a policy framework for healthy ageing. Lancet, 387:2145-54. 26520231 10.1016/S0140-6736(15)00516-4 PMC4848186

[2] Ham C (2010). The ten characteristics of the high-performing chronic care system. Health Econ Policy Law, 5:71-90. 19732475 10.1017/S1744133109990120

[3] Low LF, Yap M, Brodaty H (2011). A systematic review of different models of home and community care services for older persons. BMC Health Serv Res, 11:93. 21549010 10.1186/1472-6963-11-93 PMC3112399

[4] Eklund K, Wilhelmson K (2009). Outcomes of coordinated and integrated interventions targeting frail elderly people: a systematic review of randomised controlled trials. Health Soc Care Community, 17:447-58. 19245421 10.1111/j.1365-2524.2009.00844.x

[5] Beard JR, Officer AM, Cassels AK (2016). The World Report on Ageing and Health. Gerontologist, 56: S163-6. 26994257 10.1093/geront/gnw037

[6] Wang J, Boehm L, Mion LC (2017). Intrinsic capacity in older hospitalized adults: Implications for nursing practice. Geriatr Nurs, 38:359-61. 28778277 10.1016/j.gerinurse.2017.06.008 PMC7393792

[7] Cesari M, Araujo de Carvalho I, Amuthavalli Thiyagarajan J, Cooper C, Martin F C, Reginster J-Y, et al. (2018). Evidence for the Domains Supporting the Construct of Intrinsic Capacity. J Gerontol A Biol Sci Med Sci, 73:10-1093. 10.1093/gerona/gly011 29408961

[8] Beard JR, Jotheeswaran AT, Cesari M, Araujo De Carvalho I (2019). The structure and predictive value of intrinsic capacity in a longitudinal study of ageing. BMJ Open, 9:e26119. 10.1136/bmjopen-2018-026119 PMC6830681 31678933

[9] Beard JR, Si Y, Liu Z, Chenoweth L, Hanewald K (2021). Intrinsic Capacity: Validation of a New WHO Concept for Healthy Ageing in a Longitudinal Chinese Study. J Gerontol A Biol Sci Med Sci, 10.1093/gerona/glab226. 34343305

[10] Liu S, Yu X, Wang X, Li J, Jiang S, Kang L, et al. (2021). Intrinsic Capacity predicts adverse outcomes using Integrated Care for Older People screening tool in a senior community in Beijing. Arch Gerontol Geriatr, 94:104358. 33548677 10.1016/j.archger.2021.104358

[11] YuR, Amuthavalli ThiyagarajanJ, LeungJ, LuZ, KwokT, WooJ (2021). Validation of the Construct of Intrinsic Capacity in a Longitudinal Chinese Cohort. J Nutr Health Aging, 25:33-40.3417993810.1007/s12603-021-1637-z

[12] Charles A, Buckinx F, Locquet M, Reginster J, Petermans J, Gruslin B, et al. (2020). Prediction of Adverse Outcomes in Nursing Home Residents According to Intrinsic Capacity Proposed by the World Health Organization. J Gerontol A Biol Sci Med Sci, 75:1594-9. 31562812 10.1093/gerona/glz218

[13] Zeng X, Shen S, Xu L, Wang Y, Yang Y, Chen L, et al. (2021). The Impact of Intrinsic Capacity on Adverse Outcomes in Older Hospitalized Patients: A One-Year Follow-Up Study. Gerontology, 67:1-9. 33735899 10.1159/000512794

[14] Gonzalez-BautistaE, AndrieuS, Gutierrez-RobledoLM, Garcia-ChanesRE, de SoutoBP (2020). In the quest of a Standard Index of Intrinsic Capacity. A Critical Literature Review. J Nutr Health Aging, 24:959-65.3315562110.1007/s12603-020-1394-4

[15] Belloni G, Cesari M (2019). Frailty and Intrinsic Capacity: Two Distinct but Related Constructs. Front Med (Lausanne), 6:133. 31275941 10.3389/fmed.2019.00133 PMC6591451

[16] Woo J (2019). Frailty, Successful Aging, Resilience, and Intrinsic Capacity: a Cross-disciplinary Discourse of the Aging Process. Curr Geri Rep, 8:67-71.

[17] Gutiérrez-RobledoLM, García-ChanesRE, González-BautistaE, Rosas-CarrascoO (2021). Validation of Two Intrinsic Capacity Scales and Its Relationship with Frailty and Other Outcomes in Mexican Community-Dwelling Older Adults. J Nutr Health Aging, 25:33-40.3336746010.1007/s12603-020-1555-5

[18] Hadley EC, Kuchel GA, Newman AB, Allore HG, Bartley JM, Bergeman CS, et al. (2017). Report: NIA Workshop on Measures of Physiologic Resiliencies in Human Aging. J Gerontol A Biol Sci Med Sci, 72:980-990. 28475732 10.1093/gerona/glx015 PMC5861884

[19] Resnick B, Galik E, Dorsey S, Scheve A, Gutkin S (2011). Reliability and Validity Testing of the Physical Resilience Measure. Gerontologist, 51:643-52. 21402647 10.1093/geront/gnr016

[20] Chhetri JK, Xue QL, Ma L, Chan P, Varadhan R (2021). Intrinsic Capacity as a Determinant of Physical Resilience in Older Adults. J Nutr Health Aging, in press. 10.1007/s12603-021-1629-z PMC8035602 34545921

[21] Ma L, Chhetri JK, Zhang Y, Liu P, Chen Y, Li Y, et al. (2020). Integrated Care for Older People Screening Tool for Measuring Intrinsic Capacity: Preliminary Findings From ICOPE Pilot in China. Front Med (Lausanne), 7:576079 33330532 10.3389/fmed.2020.576079 PMC7734133

[22] Gonzalez-Bautista E, de Souto BP, Virecoulon GK, Andrieu S, Rolland Y, Vellas B (2021). Frequency of Conditions Associated with Declines in Intrinsic Capacity According to a Screening Tool in the Context of Integrated Care for Older People. J Frailty Aging, 10:94-102. 33575697 10.14283/jfa.2020.42

[23] Justice JN, Ferrucci L, Newman AB, Aroda VR, Bahnson JL, Divers J, et al. (2018). A framework for selection of blood-based biomarkers for geroscience-guided clinical trials: report from the TAME Biomarkers Workgroup. GeroScience, 40:419-36. 30151729 10.1007/s11357-018-0042-y PMC6294728

[24] Giudici KV, de Souto Barreto P, Guerville F, Beard J, Araujo De Carvalho I, Andrieu S, et al. (2019). Associations of C-reactive protein and homocysteine concentrations with the impairment of intrinsic capacity domains over a 5-year follow-up among community-dwelling older adults at risk of cognitive decline (MAPT Study). Exp Gerontol, 127:110716. 31493520 10.1016/j.exger.2019.110716

[25] MaL, ZhangY, LiuP, LiS, LiY, JiT, et al. (2020). Plasma N-Terminal Pro-B-Type Natriuretic Peptide Is Associated with Intrinsic Capacity Decline in an Older Population. J Nutr Health Aging, 25(2):271-710.1007/s12603-020-1468-333491044

[26] MaL, LiuP, ZhangY, ShaG, ZhangL, LiY (2020). High Serum Tumor Necrosis Factor Receptor 1 Levels Are Related to Risk of Low Intrinsic Capacity in Elderly Adults. J Nutr Health Aging, 25(4):416-8.10.1007/s12603-020-1533-y33786556

[27] Moll van Charante EP, Richard E, Eurelings LS, van Dalen JW, Ligthart SA, van Bussel EF, et al. (2016). Effectiveness of a 6-year multidomain vascular care intervention to prevent dementia (preDIVA): a cluster-randomised controlled trial. Lancet, 388:797-805. 27474376 10.1016/S0140-6736(16)30950-3

[28] Lehtisalo J, Levälahti E, Lindström J, Hänninen T, Paajanen T, Peltonen M, et al. (2019). Dietary changes and cognition over 2 years within a multidomain intervention trial-The Finnish Geriatric Intervention Study to Prevent Cognitive Impairment and Disability (FINGER). Alzheimers Dement, 15:410-7. 30527596 10.1016/j.jalz.2018.10.001

[29] Bae S, Lee S, Lee S, Jung S, Makino K, Harada K, et al. (2019). The effect of a multicomponent intervention to promote community activity on cognitive function in older adults with mild cognitive impairment: A randomized controlled trial. Complement Ther Med, 42: 164-9. 30670238 10.1016/j.ctim.2018.11.011

[30] van de Rest O, Geleijnse JM, Kok FJ, van Staveren WA, Dullemeijer C, Olderikkert MG, et al. (2008). Effect of fish oil on cognitive performance in older subjects: a randomized, controlled trial. Neurology, 71(6): 430-8. 18678826 10.1212/01.wnl.0000324268.45138.86

[31] Tajalizadekhoob Y, Sharifi F, Fakhrzadeh H, Mirarefin M, Ghaderpanahi M, Badamchizade Z, et al. (2011). The effect of low-dose omega 3 fatty acids on the treatment of mild to moderate depression in the elderly: a double-blind, randomized, placebo-controlled study. Eur Arch Psychiatry Clin Neurosci, 261:539-49. 21318452 10.1007/s00406-011-0191-9

[32] Sinn N, Milte CM, Street SJ, Buckley JD, Coates AM, Petkov J, et al. (2012). Effects of n-3 fatty acids, EPA v. DHA, on depressive symptoms, quality of life, memory and executive function in older adults with mild cognitive impairment: a 6-month randomised controlled trial. Br J Nutr, 107:1682-93. 21929835 10.1017/S0007114511004788

[33] Smith GI, Julliand S, Reeds DN, Sinacore DR, Klein S, Mittendorfer B (2015). Fish oil-derived n-3 PUFA therapy increases muscle mass and function in healthy older adults. Am J Clin Nutr, 102:115-22. 25994567 10.3945/ajcn.114.105833 PMC4480667

[34] Baleztena J, Ruiz-Canela M, Sayon-Orea C, Pardo M, Añorbe T, Gost JI, et al. (2018). Association between cognitive function and supplementation with omega-3 PUFAs and other nutrients in ≥ 75 years old patients: A randomized multicenter study. PloS One, 13:e193568. 10.1371/journal.pone.0193568 PMC5868762 29579102

[35] Giudici KV, de Souto Barreto P, Beard J, Cantet C, Araujo De Carvalho I, Rolland Y, et al. (2020). Effect of long-term omega-3 supplementation and a lifestyle multidomain intervention on intrinsic capacity among community-dwelling older adults: Secondary analysis of a randomized, placebo-controlled trial (MAPT study). Maturitas, 141:39-45. 33036701 10.1016/j.maturitas.2020.06.012

[36] Takeda C, Guyonnet S, Sumi Y, Vellas B, Araujo de Carvalho I (2020). Integrated Care for Older People and the Implementation in the INSPIRE Care Cohort. J Prev Alzheimers Dis, 7:70-4. 32236394 10.14283/jpad.2020.8

[37] Tavassoli N, Piau A, Berbon C, de Kerimel J, Lafont C, De Souto Barreto P, et al. (2021). Framework Implementation of the INSPIRE ICOPE-CARE Program in Collaboration with the World Health Organization (WHO) in the Occitania Region. J Frailty Aging, 10:103-9. 33575698 10.14283/jfa.2020.26

[38] de Souto Barreto P, Guyonnet S, Ader I, Andrieu S, Casteilla L, Davezac N, et al. (2020). The Inspire Research Initiative: A Program for Geroscience and Healthy Aging Research going from Animal Models to Humans and the Healthcare System. J Frailty Aging, 10(2):86-93 10.14283/jfa.2020.18 33575696

[39] Sanchez-Rodriguez D, Annweiler C, Gillain S, Vellas B (2021). Implementation of the Integrated Care of Older People (ICOPE) App in Primary Care: New Technologies in Geriatric Care during Quarantine of COVID-19 and Beyond. J Frailty Aging, 10:139-40. 33575702 10.14283/jfa.2020.24 PMC7195618

[40] Sanchez-Rodriguez D, Piccard S, Dardenne N, Giet D, Annweiler C, Gillain S (2021). Implementation of the Integrated Care of Older People (ICOPE) App and ICOPE Monitor in Primary Care: A study protocol. J Frailty Aging, 10:290-6. 34105715 10.14283/jfa.2021.22

[41] ZhouY, LiY, ZhuX, MaL (2021). Medical and Old-Age Care Integration Model and Implementation of the Integrated Care of Older People (ICOPE) in China: Opportunities and Challenges. J Nutr Health Aging, 25:720-3.3417992310.1007/s12603-021-1595-5

